# Supplementary material for: The expected behaviour of random fields in high dimensions: contradictions in the results of Bansal and Peterson (2018)
Source: Magn Reson Imaging. Author manuscript; Available in PMC 2022 Jul 1. (PMC7612853; doi:10.1016/j.mri.2021.11.010)
Supplement: Appendix [file EMS145853-supplement-Appendix.pdf]

bioRxiv preprint doi: https://doi.org/10.1101/2021.01.21.427611; this version posted April 19, 2021. The copyright holder for this preprint (which was not certified by peer review) is the author/funder, who has granted bioRxiv a license to display the preprint in perpetuity. It is made available under aCC-BY 4.0 International license.

upon. We have shown that their results disagree with our simulations which supported the existing well-established and validated theory.

4 Appendix

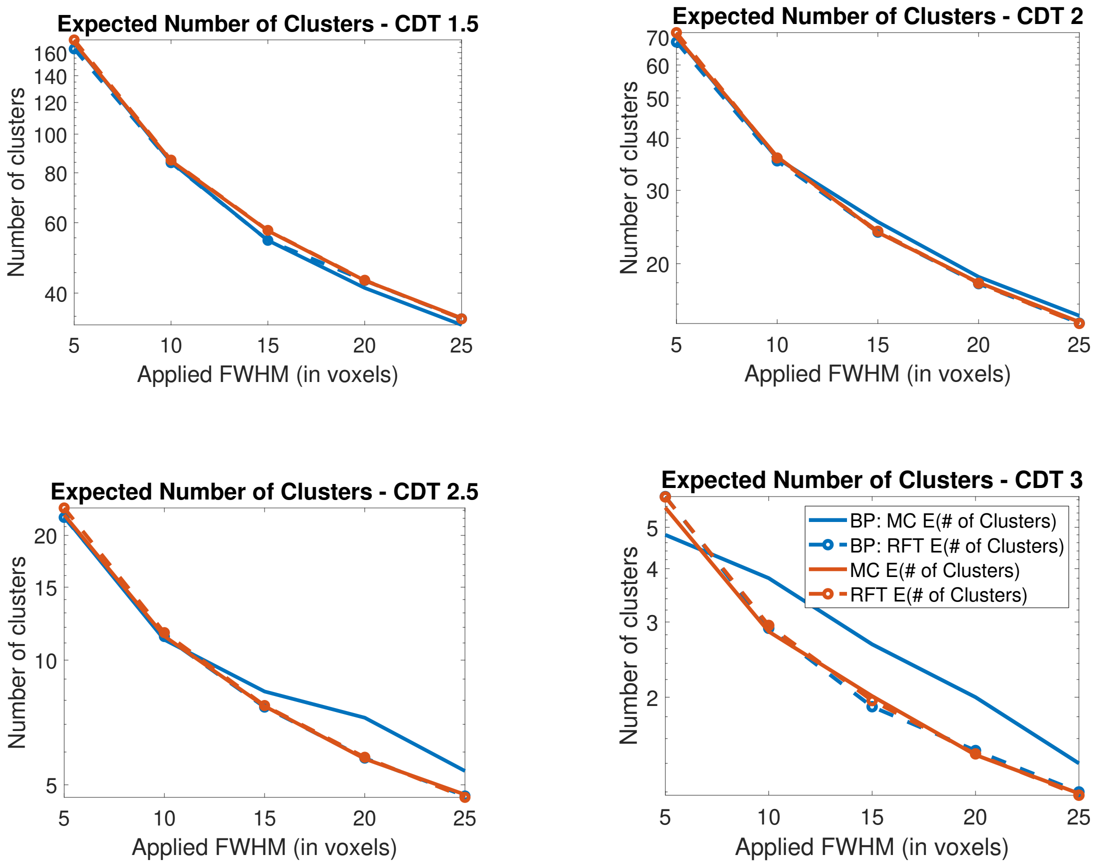

Figure 3: Comparison of Monte Carlo and theoretical expected number of clusters in 1D (in the legend # denotes the word number). At low CDTs our results match those of BP, however at high CDTs the average number of clusters that they find is slightly inflated but the theoretical number of clusters they find matches our calculations.

bioRxiv preprint doi: https://doi.org/10.1101/2021.01.21.427611; this version posted April 19, 2021. The copyright holder for this preprint (which was not certified by peer review) is the author/funder, who has granted bioRxiv a license to display the preprint in perpetuity. It is made available under aCC-BY 4.0 International license.

FWHM	Cluster Defining Threshold			
	1.5	2.0	2.5	3.0
5	$168.75 \pm 0.07(172.14)$	$69.79 \pm 0.05(71.75)$	$22.42 \pm 0.03(23.29)$	$5.59 \pm 0.01(5.89)$
10	$85.66 \pm 0.05(86.10)$	$35.62 \pm 0.04(35.89)$	$11.52 \pm 0.02(11.65)$	$2.92 \pm 0.01(2.95)$
15	$57.30 \pm 0.04(57.42)$	$23.84 \pm 0.03(23.93)$	$7.73 \pm 0.02(7.77)$	$1.95 \pm 0.01(1.96)$
20	$43.02 \pm 0.04(43.08)$	$17.92 \pm 0.03(17.96)$	$5.81 \pm 0.01(5.83)$	$1.47 \pm 0.01(1.47)$
25	$34.42 \pm 0.03(34.48)$	$14.36 \pm 0.02(14.37)$	$4.65 \pm 0.01(4.66)$	$1.17 \pm 0.01(1.18)$

(a) 1D simulations versus expected Euler characteristic.

FWHM	Cluster Defining Threshold			
	1.5	2.0	2.5	3.0
5	$218.59 \pm 0.07(222.99)$	$118.05 \pm 0.06(122.73)$	$46.59 \pm 0.04(49.51)$	$13.72 \pm 0.02(14.96)$
10	$57.34 \pm 0.03(57.95)$	$31.11 \pm 0.03(31.60)$	$12.42 \pm 0.02(12.67)$	$3.70 \pm 0.01(3.82)$
15	$26.54 \pm 0.02(26.75)$	$14.29 \pm 0.02(14.45)$	$5.69 \pm 0.01(5.77)$	$1.71 \pm 0.01(1.73)$
20	$15.48 \pm 0.02(15.61)$	$8.28 \pm 0.02(8.36)$	$3.29 \pm 0.01(3.32)$	$0.98 \pm 0.01(0.99)$
25	$10.29 \pm 0.01(10.36)$	$5.46 \pm 0.01(5.50)$	$2.15 \pm 0.01(2.17)$	$0.64 \pm 0.00(0.65)$

(b) 2D simulations versus expected Euler characteristic.

FWHM	Cluster Defining Threshold			
	1.5	2.0	2.5	3.0
5	$336.36 \pm 0.10(353.79)$	$288.06 \pm 0.08(315.00)$	$151.90 \pm 0.07(171.14)$	$55.17 \pm 0.05(64.35)$
10	$55.97 \pm 0.04(56.19)$	$44.81 \pm 0.03(45.77)$	$23.25 \pm 0.03(23.93)$	$8.46 \pm 0.02(8.80)$
15	$20.59 \pm 0.02(20.57)$	$15.47 \pm 0.02(15.61)$	$7.77 \pm 0.02(7.89)$	$2.79 \pm 0.01(2.84)$
20	$10.52 \pm 0.02(10.49)$	$7.47 \pm 0.01(7.52)$	$3.66 \pm 0.01(3.69)$	$1.29 \pm 0.01(1.31)$
25	$6.41 \pm 0.01(6.39)$	$4.35 \pm 0.01(4.36)$	$2.08 \pm 0.01(2.08)$	$0.73 \pm 0.01(0.72)$

(c) 3D simulations versus expected Euler characteristic.

Figure 4: Comparing the average number of clusters above a threshold with the expected Euler characteristic. For each table each entry is of the form $A \pm s$(EEC) where A is the average number of clusters above the threshold, $\pm s$ provides a 95% confidence interval for this average and EEC is the expected Euler characteristic provided by theory. This shows that the EEC provides a good approximation of the expected number of clusters above the threshold.

5 Acknowledgements

We would like to thank the 2 anonymous reviewers for their comments which helped to improve the quality of the manuscript.

TEN is supported by the Wellcome Trust, 100309/Z/12/Z and SD is funded by the EPSRC.

References

Robert J. Adler. *The Geometry of Random Fields*. 1981.

Robert J Adler, Jonathan E Taylor, and Keith J. Worsley. *Applications of random fields and geometry: Foundations and case studies*. 2010.
